# Effect of Structural Changes Induced by Deletion of ^54^FLRAPSWF^61^ Sequence in αB-crystallin on Chaperone Function and Anti-Apoptotic Activity

**DOI:** 10.3390/ijms221910771

**Published:** 2021-10-05

**Authors:** Sundararajan Mahalingam, Srabani Karmakar, Puttur Santhoshkumar, Krishna K. Sharma

**Affiliations:** 1 Department of Ophthalmology, School of Medicine, University of Missouri-Columbia, Columbia, MO 65212, USA; smmwt@missouri.edu (S.M.); srabani.k@technoindiaeducation.com (S.K.); 2 Department of Biochemistry, School of Medicine, University of Missouri-Columbia, Columbia, MO 65211, USA

**Keywords:** αB-crystallin, chaperone, oligomerization, apoptosis, mutant, aggregation, cataract, interactions

## Abstract

Previously, we showed that the removal of the 54–61 residues from αB-crystallin (αBΔ54–61) results in a fifty percent reduction in the oligomeric mass and a ten-fold increase in chaperone-like activity. In this study, we investigated the oligomeric organization changes in the deletion mutant contributing to the increased chaperone activity and evaluated the cytoprotection properties of the mutant protein using ARPE-19 cells. Trypsin digestion studies revealed that additional tryptic cleavage sites become susceptible in the deletion mutant than in the wild-type protein, suggesting a different subunit organization in the oligomer of the mutant protein. Static and dynamic light scattering analyses of chaperone–substrate complexes showed that the deletion mutant has more significant interaction with the substrates than wild-type protein, resulting in increased binding of the unfolding proteins. Cytotoxicity studies carried out with ARPE-19 cells showed an enhancement in anti-apoptotic activity in αBΔ54–61 as compared with the wild-type protein. The improved anti-apoptotic activity of the mutant is also supported by reduced caspase activation and normalization of the apoptotic cascade components level in cells treated with the deletion mutant. Our study suggests that altered oligomeric assembly with increased substrate affinity could be the basis for the enhanced chaperone function of the αBΔ54–61 protein.

## 1. Introduction

α-Crystallin, a small heat shock protein family member, is the most abundant protein in vertebrate eye lenses and is responsible for maintaining lens transparency [1,2]. It has two subunits, A and B, with a molecular mass of ~20 kD, and the sequence homology between the two subunits is about 56%. The two subunits exist as spherical hetero-oligomers of 400–800 kDa mass range. αB-crystallin, the less abundant α-crystallin subunit in the lens, is also present in other organs in lesser quantities [3] and has anti-apoptotic properties [4]. αB-crystallin is also linked to some protein aggregation diseases, such as myopathies and neuropathies [5,6,7]. Each α-crystallin subunit has three structural regions, the central conserved α-crystallin domain (ACD) of about 90 residues flanked by an N-terminal domain of approximately 60 residues and a C-terminal extension of 25 residues containing the IXI motif. While the N-terminal domain is considered essential for subunit–subunit interactions and quaternary structural arrangement [8], the C-terminal extension with a higher polar amino acid content is responsible for the soluble nature of the protein [9]. Recently, biophysical techniques, such as solid-state NMR and SAXS, also proved that ACD is responsible for dimer contact points, and the N-terminal domain and C-terminal IXI motif are essential for the higher order oligomerization of mammalian sHSPs [10,11,12]. αB-crystallin can exist as an oligomeric protein that is highly dynamic in the organization of its subunits, wherein 24–32 subunits can coexist and exchange rapidly [13]. A recent study determined the structure of the variable N-terminal domain of αB-crystallin and identified two beta strands that exist in multiple structural environments [14].

**Figure 1:**
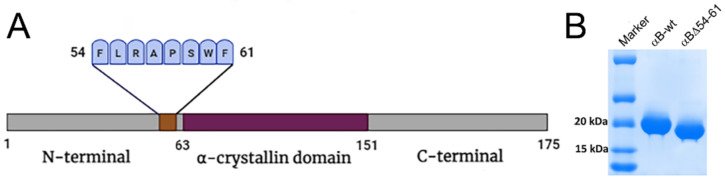
(**A**) Schematic representation of αB-crystallin showing central α-crystallin domain flanked by N-terminal domain and C-terminal domain and deleted 54–61 sequence. (**B**). SDS-PAGE profile of overexpressed and purified αB-wt and αBΔ54–61.

Several studies have established the involvement of the N-terminal region of small heat shock proteins in providing contact points for subunit–subunit interactions [14,15,16,17]. A complete deletion of the N-terminal domain from α-crystallin resulted in tetrameric structure formation. The oligomeric association likely provides additional strength to the folded subunit structure. Our studies have shown that amino acid sequences 42–57, 60–71, and 88–123 of αB-crystallin are interacting regions with αA-crystallin during hetero-oligomer formation [18,19]. To understand the role of the N-terminal domain in the structure function of αB-crystallin, we deleted 54–61 residues of the protein (Figure 1A). This deletion resulted in smaller oligomers with fewer subunits in each oligomer but with significantly higher chaperone activity function [20]. Ghosh et al. [21,22] reported that the 43–58 sequence of αB-crystallin is one of the substrate interaction sites during chaperone function and one of the αA-crystallin interacting sites. Nagaraj et al. [23] studied the paradoxical effects of deletion and substitution of R56 of αB-crystallin on its chaperone function. In other studies, it has been proposed that phosphorylation sites in the N-terminal region provide a crucial switch for conformational changes and regulation of the activity of α-crystallins [24,25].

The phosphorylation of αB-crystallin in vivo under stress and pathological conditions has been reported [25,26,27]. Serine 59 is one of the three phosphorylation sites in αB-crystallin, and it is part of the deleted sequence of the mutant of this study. Numerous studies have shown that α-crystallin has anti-apoptotic properties and can protect cells from a range of stress conditions induced by hydrogen peroxide, staurosporine, UV-A light, tumor necrosis factor, and etoposide [28,29,30]. It can associate with pro-apoptotic molecules, such as p53, Bax, and Bcl, and prevent their translocation to mitochondria during apoptosis [31]. A previous study has reported that the chaperone function and anti-apoptotic activity of αA-crystallin are interrelated [32]. Given that there is such a diverse role for αB-crystallin, this study was undertaken to understand the consequence of deleting the 54–61 region of αB-crystallin in the N-terminal domain function. We investigated the protection of stressed cells from apoptosis in the presence of the deletion mutant and compared the results obtained with the wild-type protein and found that the deletion mutant has higher anti-apoptotic activity. Additionally, the results show that the deletion mutant shows increased binding of unfolding proteins, indicating that the formation of smaller sized oligomers did not compromise the protein’s biological activity.

## 2. Results

### 2.1. The Increase in Chaperone Activity of αBΔ54–61 Mutant Varies with the Substrate Protein

**Figure 2:**
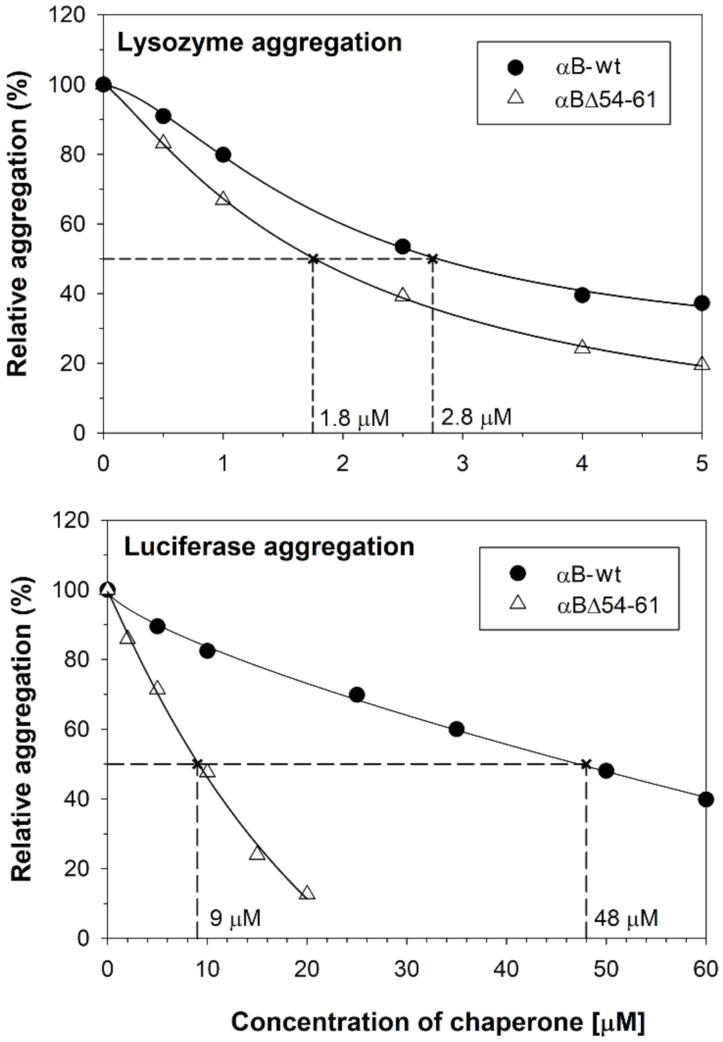
Chaperone-like activity assays of αB-wt and αBΔ54–61. Relative aggregation of lysozyme (upper panel) and luciferase (lower panel) in PBS in the presence of different concentrations of chaperone proteins was estimated by monitoring the light scattering at 360 nm on a plate reader. The result shown is representative of three independent experiments. The EC50 (effective chaperone protein concentration required to suppress the substrate protein aggregation by 50%) values are shown in the figure. The substrate protein aggregation (scattering at 360 nm) in the absence of chaperone protein is considered 100% aggregation. The aggregation of substrate protein shown at each concentration of chaperone protein tested is relative to the aggregation of substrate protein without the chaperone protein.

We reported earlier that deletion of the 54–61 amino acid residues from αB-crystallin results in a smaller homo-oligomer with a ten-fold increase in chaperone activity against alcohol dehydrogenase (ADH) and citrate synthase (CS) substrates [20]. In this study, we compared the chaperone activities with two additional substrates (lysozyme and luciferase) to assess if the enhanced chaperone function of the deletion mutant is substrate dependent. The deletion mutant showed higher anti-aggregation activity compared to the wild-type protein with both the substrates. The fold increase in anti-aggregation activity of the deletion mutant varied with the substrate protein. The αBΔ54–61 mutant protein was 1.5- and 5.3-fold more potent than αB-wt in suppressing the aggregation of 10 µM lysozyme and 1 µM luciferase, respectively. While 1.8 µM of αBΔ54–61 decreased lysozyme aggregation by 50% to achieve similar suppression, 2.8 µM for αB-wt crystallin (Figure 2 upper panel) was needed. When luciferase was used as a client protein, 9 µM of αBΔ54–61 was required to suppress the aggregation by 50%, whereas 48 µM of αB-wt was needed to provide the same level of suppression (Figure 2 lower panel).

### 2.2. αBΔ54–61 Is More Susceptible to Cleavage by Trypsin

**Figure 3:**
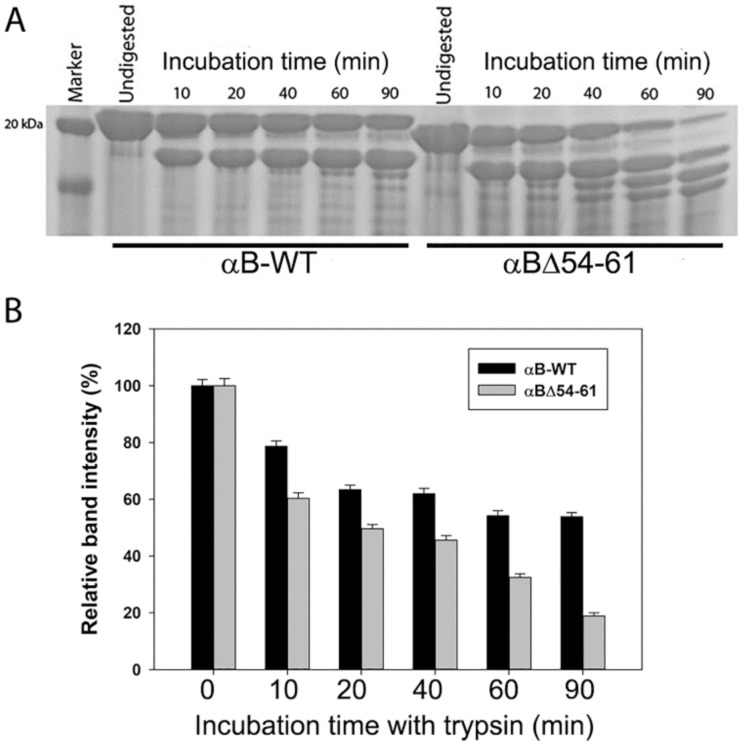
(**A**) SDS-PAGE analysis of incubation mixtures of αB-wt and αBΔ54–61 with trypsin analyzed at different time points. Protein samples were incubated with trypsin (1:200 protease to protein w/w) at 37 °C. (**B**) Bar graph showing a decrease in full-length protein band intensities against the incubation time. The SDS-PAGE gels were imaged on a BioRad ChemiDoc XRS+ imaging system (Bio-Rad laboratories, Hercules, CA, USA), and the band intensities were analyzed using Image Lab software (Bio-Rad). The data shown are the mean + SD of three independent experiments.

The treatment of the wild-type and the deletion mutant proteins with trypsin showed that αBΔ54–61 was more readily cleaved by the protease than that of wild-type αB-crystallin, suggesting that the deletion of the 54–61 region induces a conformation change in the protein sufficient enough to expose several trypsin-susceptible peptide bonds in the subunits. SDS-PAGE analysis of trypsin-treated αBΔ54–61 showed >80% cleavage of the protein band at the ~20 kDa region in 90 min. In contrast, about 58% of the wild-type protein band (~20 kDa) remained intact under similar experimental conditions (Figure 3A,B). The trypsin treatment of αBΔ54–61 generated truncated proteins of 5–17 kDa in 90 min. This pattern of cleavage of αB-wt was not observed during the 90 min of digestion with trypsin. Mass spectrometric analysis of αB-wt digested with trypsin for 60 min showed cleavage of the ^157^Arg-Thr^158^ bond, whereas in αBΔ54–61, ^11^Arg-Arg^12^, ^22^Arg-Leu^23^, ^69^Arg-Leu^70^, and ^157^Arg-Thr^158^ were the most susceptible peptide bonds under similar conditions (the residue numbering reflects the position in the wild-type protein).

### 2.3. Deletion Mutant Forms Chaperone–Substrate Complex with Higher Mass

**Figure 4:**
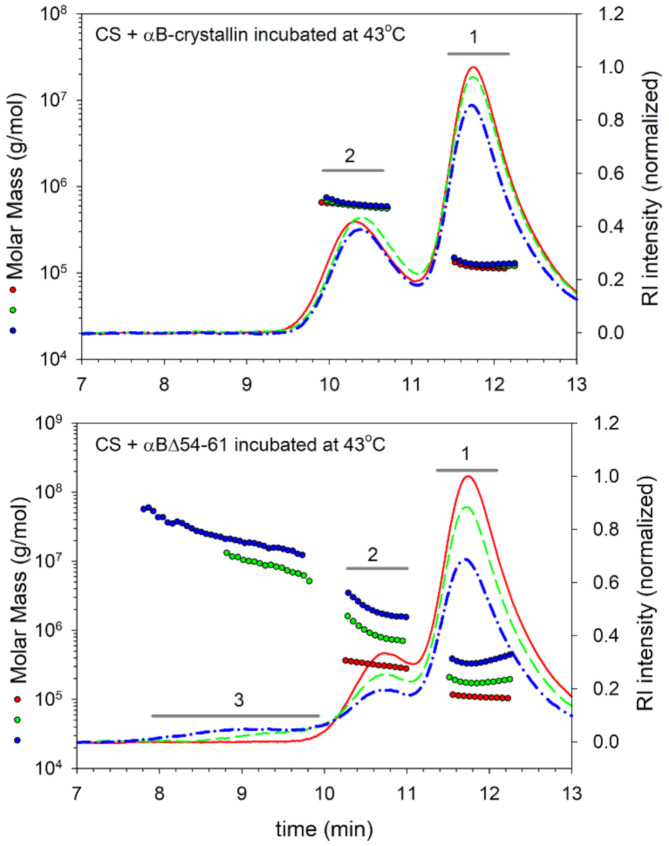
Refractive index profiles of chaperone–substrate complexes showing the molar mass distribution across the peaks. MALS analysis of αB-wt or αBΔ54–61 and CS incubation mixtures at different time points: red line—0 min, green line—40 min, blue line—80 min incubation. The **upper panel** shows the molar mass distribution in the incubation mixtures of αB-wt and CS. The **lower panel** shows molar mass analysis of αBΔ54–61 and CS after incubation. Other experimental details are included under the Methods section.

We evaluated the interaction of chaperone proteins with CS at 43 °C by analyzing the incubation mixtures at different time intervals on an HPLC coupled to a multi-angle light scattering (MALS) detector. Analysis of the αB-wt + CS mixtures before incubation (0 min) showed two peaks: one eluting at 11.8 min (peak 1) for CS and the other one eluting at 10.3 min (peak 2) for αB-wt (Figure 4 upper panel). With the αBΔ54–61 + CS mixtures, the peaks were seen eluting at 11.8 and 10.7 min for CS and αBΔ54–61, respectively, at 0 min (Figure 4, lower panel). After 40 and 80 min of incubation at 43 °C, there was no significant shift in the elution time point of the peaks or a change in peak intensities in samples with αB-wt. In contrast, samples with αBΔ54–61 showed a substantial reduction in peak 1 and peak 2 heights, and an additional peak appeared between 7.8 and 9.8 min (peak 3), suggesting the formation of large complexes. The refractive index (RI) profiles showed that <7.5% of the total protein mixture eluted in the peak 3 region. The elution profile of αB-wt and the citrate synthase reaction mixture incubated at 43 °C did not show a complex peak eluting in the 7.8–9.8 min region (Figure 4, upper panel). The molar mass (Mw) and hydrodynamic radii (Rh) estimated from the light scattering detector signals using ASTRA 6.1 software for the different protein peaks identified in Figure 4 are summarized in Table 1.

The Mw and Rh of proteins in peaks 2 and 3 increased to a greater extent in the αBΔ54–61 + CS incubation mixtures with time. In peak 3 of the αBΔ54–61 + CS sample, oligomers with an average mass of 8105 kDa were seen after 40 min incubation, and in the 80 min incubated sample, the average mass of the oligomers increased to 21,000 kDa (Table 1). The third peak’s mass fraction also rose from 3.8% at 40 min to 7.5% at 80 min, showing a time-dependent binding process leading to the formation of larger aggregates reflecting the increased unfolding protein binding capacity of αBΔ54–61. When incubated under identical conditions, the mutant protein by itself did not show any change in mass (data not shown). In contrast to αBΔ54–61, αB-wt did not show an increase in Mw or Rh of peak 2. The RI profiles show an interaction between αB-wt and CS proteins. However, the interactions are minimal and do not lead to a significant increase in the Mw of αB-wt. It is plausible that αB oligomers might have reorganized to accommodate the unfolding substrate and resist the formation of large complexes.

**Figure 5:**
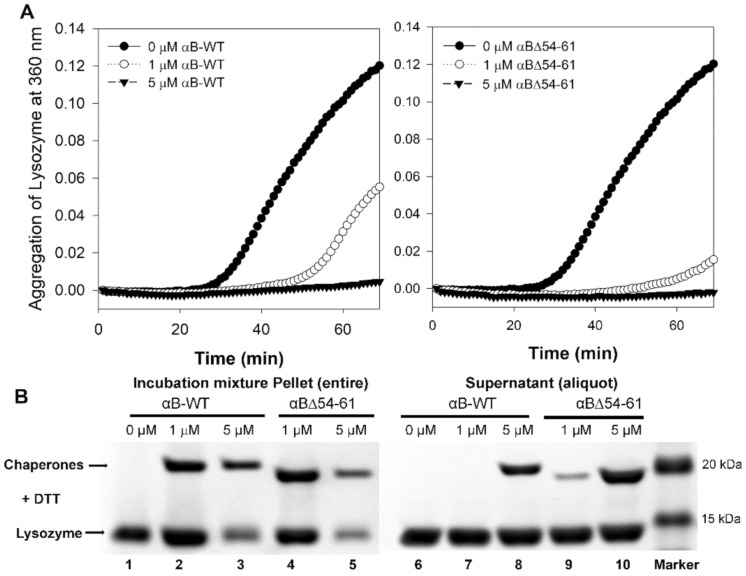
(**A**) Comparison of chaperone activity of αB-wt and αBΔ54–61 at two equimolar concentrations (1 and 5 µM) using lysozyme (5 µM) as the aggregation substrate. (**B**) SDS-PAGE of the chaperone assay supernatant and pellet fractions analyzed at the end of the assay. The αBΔ54–61 has a higher substrate binding capacity than that of αB-wt.

To test if the increased chaperone activity of the deletion mutant is due to its enhanced substrate binding, we carried out a separate experiment using lysozyme (5 µM) as the aggregating substrate. The aggregation of lysozyme was carried out in 0.25 mL PBS at 37 °C for 60 min in the presence or absence of (1 or 5 µM) αB-wt or αBΔ54–61. When used at 1 µM, αB-wt showed a 54% and the αBΔ54–61 showed an 87% reduction in the aggregation of lysozyme (Figure 5A). At a 1:1 molar ratio to lysozyme, both crystallins suppressed substrate aggregation by >95%. At the end of the chaperone assay, the samples were centrifuged at 6000 rpm for 20 min to separate the aggregates. The volume of the sample supernatants was adjusted to 0.25 mL, and an aliquot was run on SDS-PAGE along with the sample precipitates to compare the band intensities. The chaperone proteins and the lysozyme mixtures without the DTT and with and without incubation were also processed simultaneously. The SDS-PAGE gels were imaged on a BioRad ChemiDoc XRS+ imaging system, and the band intensities were analyzed using Image Lab software. The SDS-PAGE images of the incubation mixture pellet and an aliquot of the supernatant after adjusting the volume to 0.25 mL are shown in Figure 5B. After normalizing the band intensity, we estimated that 5% of the lysozyme precipitated in the samples without chaperone proteins at the end of the incubation (lane 1). The amount of lysozyme in the sediments containing 1 µM αB-wt (lane 2) increased to 7%. The presence of a strong band for αB-wt in lane 2 and the absence of a band for the chaperone protein in the supernatant (lane 7) suggest that all the αB-wt oligomers participated in the chaperone action and that the complexes precipitated upon centrifugation, resulting in an increased band intensity for lysozyme when compared to the control (lane 2 vs. lane 1). In the incubation mixtures containing 5 µM αB-wt, despite >95% suppression of the substrate protein aggregation, a 3% precipitation of lysozyme was seen (lane 3), which could be due to the settling of some of the complexes during centrifugation. Compared to the incubation mixtures with αB-wt, more lysozyme was retained in the supernatant in the samples with αBΔ54–61. The band intensities of the sediments showed 4.2 and 1.5% lysozyme with samples containing 1 µM (lane 4) and 5 µM (lane 5) αBΔ54–61, respectively. The band intensities of the chaperone protein in these lanes again suggest that a significant portion of the lysozyme precipitated as a complex during centrifugation despite the incubation mixture remaining transparent at the end of the assay. The data in total suggest that αBΔ54–61 has a higher capacity to interact with unfolding proteins in solution and that the interaction of substrate proteins occurs at the oligomeric level rather than with individual chaperone subunits.

### 2.4. Deletion Mutant Showed Greater Anti-Oxidative and Anti-Apoptotic Activity Than αB-wt

**Figure 6:**
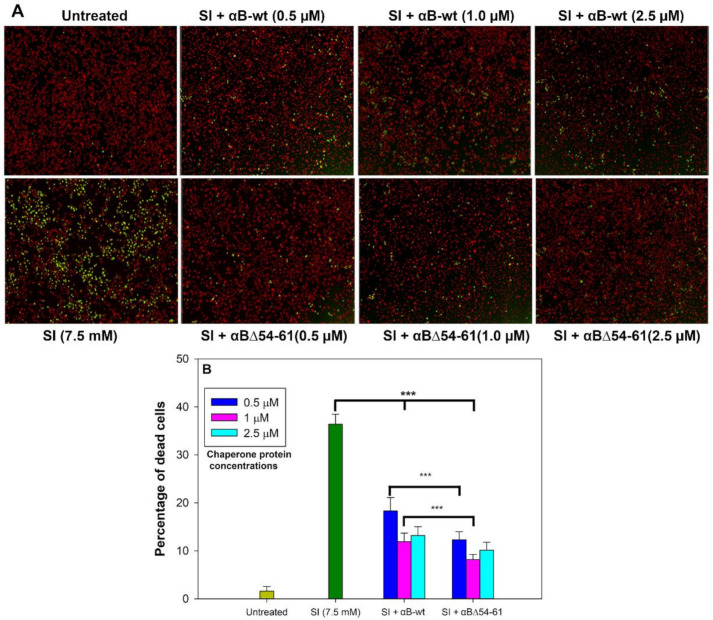
Anti-apoptotic activity of αB-wt and αBΔ54–61 against SI-induced apoptosis in ARPE-19 cells. (**A**) Serum-starved ARPE-19 cells were simultaneously treated with 0.5–2.5 µM proteins and 7.5 mM sodium iodate for 24 h as described under Methods. Cytotoxicity was measured using an EarlyTox cell integrity assay kit from Molecular Devices, San Jose, CA, USA as described under Methods. The cells were imaged on a SpectraMax MiniMax 300 Imaging Cytometer (Molecular Devices) equipped with a single 4X objective. (**B**) Bar diagram showing the percentage of dead cells calculated after live/dead cell imaging using SoftMax Pro software (Molecular Devices). The data shown are an average of six analyses performed on images captured from different wells. The asterisks (***) indicate a *p*-value < 0.005 (*n* = 6).

ARPE-19 cells were used to examine whether the deletion of one of the critical phosphorylation sites (S59) in αBΔ54–61 retained the anti-oxidative and anti-apoptotic activities of αB-wt [33]. The cells were treated with sodium iodate (SI) to induce oxidative insult and cytotoxicity [34]. The ARPE-19 cells treated with 7.5 mM of SI for 24 h showed 36.3 ± 0.1% dead cells (Figure 6A,B). In the presence of αB-wt (0.5 µM), SI-induced cell death was reduced to 18.3 ± 0.4%, whereas at 1.0 µM of αB-wt, the dead cells were 11.9 ± 0.3%. These values are equal to 50 and 68% protection of SI-susceptible cells by αB-wt. However, in the presence of αBΔ54–61 (0.5 µM), SI-induced cell death was reduced to 12.3 ± 0.9% and at twice the concentration of αBΔ54–61 (1.0 µM) to 8.2 ± 0.6% (Figure 6B), which is equal to 66 and 77% protection, a significantly (*p* < 0.005) higher protection compared to αB-wt. Increasing the chaperone protein concentration to 2.5 µM in the assay did not further increase the protection of APRE-19 cells from SI.

**Figure 7:**
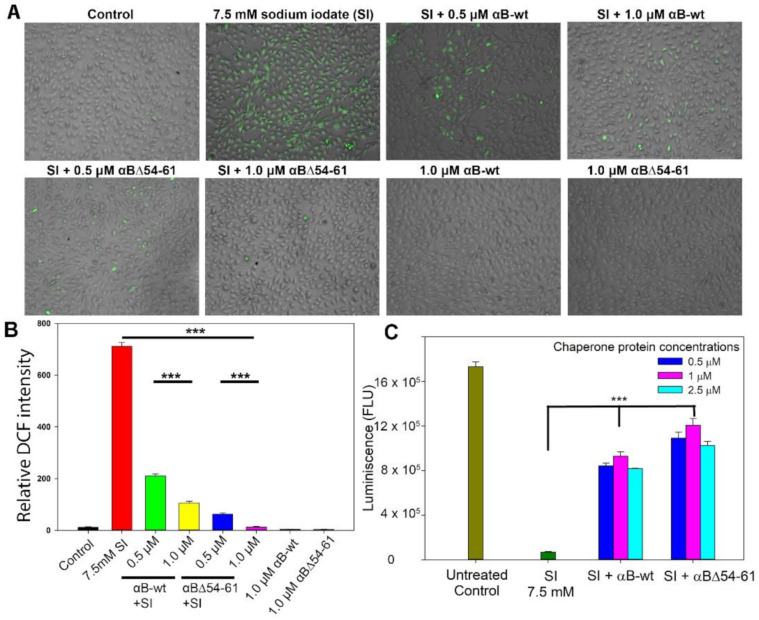
(**A**). Anti-oxidative potentials of αB-wt and αBΔ54–61 in SI-treated ARPE-19 cells. ARPE-19 cells cultured on a 96-well plate were treated with SI and/or αB-crystallins for 24 h. The SI-induced reactive oxygen species (ROS) generation was measured by 2′,7′-Dichlorofluorescin diacetate (DCFH-DA) staining. The images were captured in EVOS FL Auto2 Imaging System (Thermo Fisher Scientific, Waltham, MA, USA) with 10× magnification. (**B**) The relative 2’-7’-dichlorofluorescein (DCF) intensity (green) calculated using EVOS™ image analysis software (version 1.4.998.659). (**C**) ARPE-19 cells cultured on a 96-well plate were treated with SI and/or αB-wt and αBΔ54–61 for 24 h. CellTiter Glo 2.0 assay was used to measure ATP, an indicator of cell health. ATP is quantified indirectly by measuring the luminescence of the cells after treatment and is expressed as relative light units (RLU/well). The data shown are an average of six analyses performed on images captured from different wells. The asterisks (***) represent a *p*-value < 0.005.

The anti-oxidative property of the crystallins was also measured by estimating the intracellular ROS level in ARPE-19 cells treated with 7.5 mM SI and αB-wt or αBΔ54–61. As shown in Figure 7A,B, the SI treatment of ARPE-19 cells results in ROS release. We observed that the treatment of SI-challenged cells with 0.5 and 1.0 µM αB-wt decreases ROS production by 70 ± 3.4 and 85 ± 0.6%, respectively. In contrast, under similar experimental conditions, ROS production in SI-treated cells reduces by 93 ± 2 and 98 ± 1% when 0.5 and 1.0 µM αBΔ54–61-crystallin is included in the culture. At both concentrations tested, αBΔ54–61 showed higher ROS inhibitory activity than αB-wt. The difference was statistically significant (*p*< 0.005).

Favorable results were obtained when the ARPE-19 cells treated with SI and cultured in the presence of αB-wt or αBΔ54–61 were subjected to a cell viability assay using CellTiter Glo 2.0 (Promega, Madison, WI, USA) (Figure 7C). Analysis of the cell viability assay showed that the inclusion of αB-wt or the deletion mutant during cell culture resulted in significant protection of ARPE-19 cells from SI. The protection offered by the deletion mutant was consistently higher than that shown by αB-wt when similar concentrations of proteins were tested.

### 2.5. The Increased Anti-Apoptotic Activity of αBΔ54–61 Was Associated with Reduced Caspase Activation

**Figure 8:**
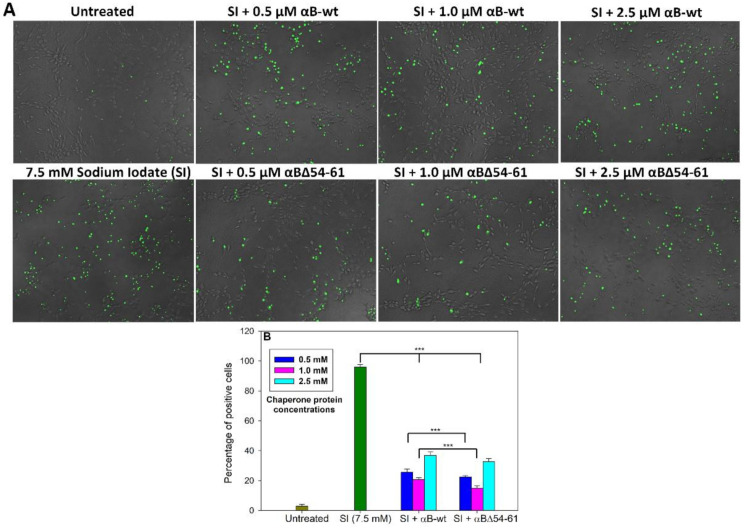
Effect of αB-crystallin sequence 54–61 deletion on caspase activation. (**A**) ARPE-19 cells treated with sodium iodate and/or αB-crystallins and the relative amount of caspase activation was determined after 24 h using NucView 488 Caspase-3/7 assay kit as described under Methods. The images were captured using EVOS FL Auto2 Imaging System in 4× magnification. (**B**) The percentage of positive cells in each sample was calculated from the cell imaging data using EVOS™ image analysis software (version 1.4.998.659) normalized with the total number of cells in each well. The data show an average of six analyses performed on images captured from different wells for caspase activity in cells simultaneously treated with sodium iodate and αB-crystallins. The asterisks (***) represent a *p*-value < 0.005.

Increased caspase activation is one of the hallmarks of the apoptosis process. Therefore, we tested the ARPE-19 cells treated with 7.5 mM of SI and αB-wt or αBΔ54–61 for caspase-3/7 activation using NucView 488 Caspase-3 assay kit (Biotium, Inc., Fremont, CA, USA). ARPE-19 cells challenged with SI in the presence and absence of chaperone proteins for 24 h showed that both αB-wt and αBΔ54–61 at the 0.5 µM level reduced SI-induced cell death, where the crystallins inhibited caspase-3/7 activation by 74 and 78%, respectively (Figure 8A,B). Furthermore, cells treated with αBΔ54–61 (0.5 and 1.0 µM) showed a 12–18% higher reduction in caspase activation compared to the αB-wt protein, suggesting that the deletion of the 54–61 residues in αB-crystallin leads to an enhanced suppression of caspase activation during stress.

### 2.6. Treatment with αB-wt and αBΔ54–61 Alters the Levels of Apoptotic Cascade Components in SI-Treated ARPE-19 Cells

**Figure 9:**
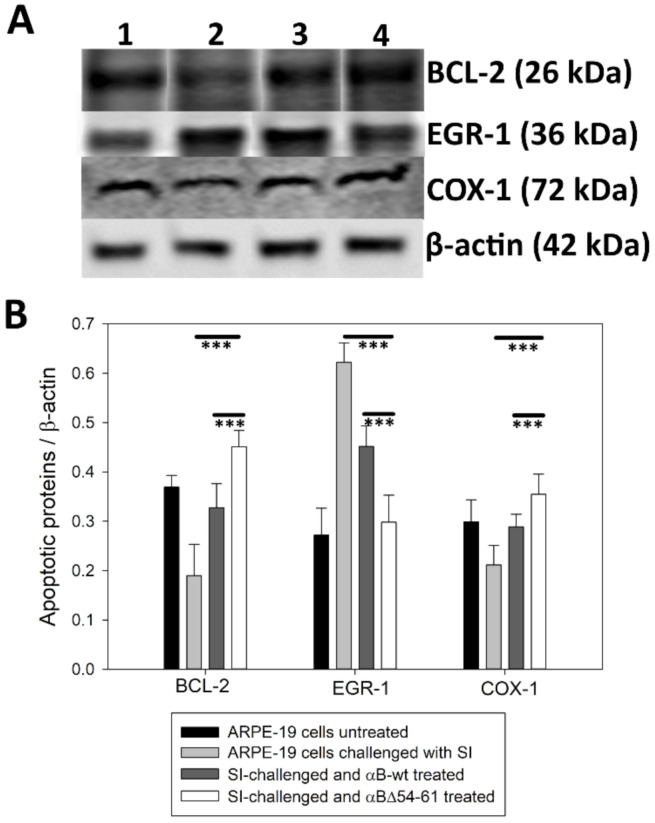
Immunoblot showing the intensity of apoptotic cascade components. (**A**) Western blotting analysis showing the intracellular expression of apoptotic cascade components in experimental groups of ARPE-19 cells. (1) ARPE-19 cells untreated, (2) ARPE-19 cells treated with 7.5 mM SI, (3) ARPE-19 cells simultaneously treated with 7.5 mM SI and 1µM of αB-wt, (4) ARPE-19 cells simultaneously treated with 7.5 mM SI and 1µM of αBΔ54–61. (**B**) Bar graphs of proteins of Bcl-2, EGR-1, and COX-1 normalized to cellular β-actin protein levels. The asterisks (***) indicate a *p*-value < 0.005.

SI is a widely used oxidant for generating oxidative damage-associated death of retinal pigment epithelium (RPE) cells [34]. The generation of ROS causes cell death via apoptosis [35]. The expression of the apoptotic cascade components, such as early growth response protein-1 (EGR-1), cytochrome c oxidase I (COX-I), and B-cell chronic lymphoma 2 (Bcl-2) protein, was investigated by an immunoblot analysis of proteins extracted from ARPE-19 cells treated with αB-wt or αBΔ54–61 for 24 h. The results shown in Figure 9 suggest that αB-crystallin proteins provide protection against apoptotic cell death in stress-induced ARPE-19 cells by regulating EGR-1, COX-1, and Bcl-2 expression levels (Figure 9A,B). Krishna K. SharmaBcl-2 suppresses apoptosis in a variety of cell systems by controlling the mitochondrial membrane permeability [36]. Therefore, maintaining its normal level is essential for the survival of cells. When ARPE-19 cells were treated with SI, we observed a decrease in the Bcl-2 level. Adding αB-wt restored the Bcl-2 level comparable to that in cells that were not exposed to SI. In cells exposed to αBΔ54–61 and SI, the Bcl-2 level was significantly higher than that of the cells exposed to αB-wt and SI.

Previous studies have shown that oxidative stress-induced cell death is associated with the upregulation of EGR-1 [37]. We examined whether EGR-1 is modulated by αB-wt and αBΔ54–61 in ARPE-19 cells cultured in the presence of stress inducer, SI. As shown in Figure 9A,B, there was an increase in EGR-1 immunoreactivity in SI-treated ARPE-19 cells, and the addition of αB-wt or αBΔ54–61 attenuated this. Additionally, we found that αBΔ54–61 was more effective than αB-wt in suppressing oxidative stress-induced enhancement of EGR-1 protein.

Changes in the COX-1 gene expression level are related to cellular stress and apoptosis [37]. Studies have shown that lenses subjected to selenite-induced oxidative stress show decreased COX-1 transcripts. Here, we observed a decrease in COX-1 reactivity during a Western blot of SI-treated ARPE-19 cell extracts as shown in Figure 9A,B. However, the co-treatment of ARPE-19 cells with SI and αB-wt or αBΔ54–61 resulted in restoring COX-1 reactivity to the same level of that in control ARPE-19 cells.

### 2.7. Transduction of αB-wt and αBΔ54–61 into ARPE-19 Cells

**Figure 10:**
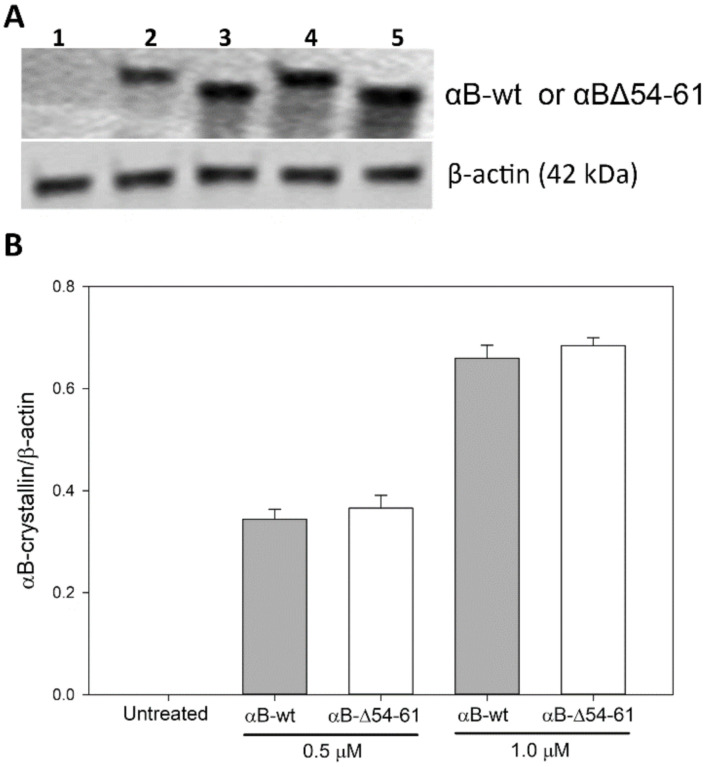
(**A**) Western blotting analysis showing the crystallin proteins transduced into the ARPE-19 cells. Lanes—(1) ARPE-19 cells untreated, (2) 0.5 μM of αB-wt, (3) 0.5 μM of αBΔ54–61, (4) 1 μM of αB-wt, and (5) 1μM of αBΔ54–61. (**B**) Bar graphs of normalized Western blot band intensities.

A comparative study of αB-wt and αBΔ54–61 in the cell culture system showed that the deletion mutant has superior anti-apoptotic and anti-oxidative properties. We tested if the enhanced activity of the mutant protein is due to its increased transduction into cells. Immunoblot analysis of lysed ARPE-19 cells treated with the two proteins did not show any significant difference in the amount of protein taken up by the cells (Figure 10). Therefore, we conclude that both αB-wt and αBΔ54–61 proteins entered the cells equally, and the mutant protein offered maximum protection from sodium iodate-induced oxidative stress and cell death compared to that observed with αB-wt. An increase in the cytoprotective effect observed with αBΔ54–61 is a gain of function after deleting a specific sequence in the parent protein.

## 3. Discussion

Previous studies concluded that the N-terminal domain in α-crystallin subunits plays a role in forming homo- or hetero-oligomers [1,14,38,39]. The crystal structure of the sHSP 16.5 showed that the amino acid residues in the N-terminal domain (NTD) are highly disordered and unresolved in oligomers [40]. Earlier work has established that deleting the total NTD from αA-crystallin leads to an extreme reduction in the oligomeric size, from 800 to 60 kDa. Still, this deletion does not affect the subunit exchange [41]. Peptide scan studies indicated that the residues 42–57 and 60–71 of αB-crystallin are essential for interaction with αA-crystallin [21]. Another study has reported that the residues 43–57 of NTD of human αB-crystallin play a significant role in chaperone activity and subunit assembly [22]. α-Lactalbumin binding studies showed that the 25–47 region in the NTD of αB-crystallin might be one of the substrate-binding regions [42]. Crosslinking studies involving ADH and αB-crystallin revealed that the ^57^APSWIDTGLSEMR^69^ region in αB-crystallin is involved in chaperone activity [43]. Ghosh et al. have reported that the ^43^SLSPFYLRPPSFLRAP^58^ sequence in αB-crystallin has a dual role—interaction with αA-crystallin and chaperone function [22]. The proteolytic digestion of a reduced α-lactalbumin–αB-crystallin complex suggested that the 25–47 region of αB-crystallin likely encompasses some residues that interact with the client proteins during chaperone function [42]. Thus, based on multiple studies, it appears that the NTD region plays a critical role in protein oligomerization, chaperone function, and subunit interaction in αB-crystallin, although evidence for a direct interaction of specific residue(s) in the NTD with client proteins is still lacking.

Our earlier study showed that the deletion of the amino acid residues 54–61 from the N-terminal domain of αB-crystallin improves in vitro chaperone function by as much as 10-fold when unfolding ADH is used as a substrate [20]. When the heat-induced unfolding of luciferase was initiated at 37 °C, a 4.5-fold enhanced chaperone activity was observed with αBΔ54–61 compared to that of the WT protein (Figure 2, lower panel). Compared to αB-wt, αBΔ54–61 was also more effective in suppressing the aggregation of the lysozyme unfolded by EDTA (Figure 2, upper panel), but the relative increase in chaperone activity was less than 2.0-fold. Thus, it appears that the substrate-dependent variation in chaperone activity shown by αB-wt is also observed with αBΔ54–61. During DLS analysis of the reaction mixtures of αB-wt + CS and αBΔ54–61 + CS, we found that the enhanced chaperone activity of αBΔ54–61 results in a time-dependent greater binding of unfolding CS to αBΔ54–61 to form larger complexes (peak 3 in Figure 4, lower panel) with a higher hydrodynamic radius (Table 1). Similar changes were not observed when the αB-wt- and CS-containing reaction mixtures were analyzed (Figure 4, upper panel). Therefore, the chaperone assay results (Figure 2) and DLS analysis data of the chaperone assay mixtures (Figure 4 and Table 1) demonstrate that αBΔ54–61, which forms a smaller oligomer, has an increased capacity to bind unfolding proteins compared to αB-wt.

In an earlier study, when a 14 amino acid sequence was inserted at the N-terminal domain of Hsp 16.5 to result in Hsp 16.5 P1, a larger oligomer was formed and showed increased binding of client proteins [44]. That observation was interpreted as a gain of chaperone activity due to the activation of Hsp 16.5 after insertion of the 14 residue sequence. In a subsequent study, the authors reported a positive correlation between the oligomer size and higher chaperone efficiency when the insertion sequence was altered to generate a series of oligomers [45]. Unlike the observations made with Hsp 16.5 P1 variants, our study shows increased chaperone activity in smaller oligomers formed by αBΔ54–61 when compared to αB-wt. Our observation is also contrary to other reports that stated decreased chaperone activity when the oligomer size was decreased due to modifications or mutations in sHsps [46]. The enhanced chaperone activity in αBΔ54–61 observed during this study was less than that observed with αB-crystallin immobilized on a solid surface [47]. Solid surface immobilization apparently increased the chaperone activity of αB-crystallin by 100- to 5000-fold. The molecular mechanisms contributing to a 100- to 5000-fold increase in chaperone activity are yet to be understood. The significant increase in anti-aggregation activity (2- to 10-fold) that we observed with αBΔ54–61 is likely due to the re-organization of the oligomers and the exposure of the cryptic hydrophobic site. This view is consistent with the previously published results relating to the structural perturbation of α-crystallin and enhanced chaperone function [48]. Previous studies have shown that heat treatment increases the chaperone activity of α-crystallins [49], and this enhanced chaperone activity was attributed to the partial unfolding of the protein and the exposure of ‘hidden’ chaperone sites. The observation of an increase in the hydrophobic probe bis-ANS binding to αBΔ54–61 [20] and the increased trypsin susceptibility of αBΔ54–61 compared to αB-wt are in support of molecular rearrangement in the subunits that form the oligomer.

Rearrangement of the subunits in αBΔ54–61 is further supported by the susceptibility of the deletion mutant to trypsin compared to αB-wt (Figure 3). Unlike the αB-wt protein that showed one predominant trypsin cleavage site in the C-terminal extension (^157^Arg-Thr^158^), αBΔ54–61 was cleaved at two sites (^11^Arg-Arg^12^ and ^22^Arg-Leu^23^) in the N-terminal domain and at the beginning of the conserved α-crystallin domain (^69^Arg-Leu^70^) and C-terminal extension (^157^Arg-Thr^158^). These results suggest that the 54–61 region in αB-crystallin plays a role in stabilizing the oligomer. Additional support for this view comes from the studies carried out with the Arg 56 mutant of αB-crystallin, where it was found that the mutation influenced protein structure, chaperone function, and its interactions with αA-crystallin [23]. Further, it has been reported that R56 is critical for subunit interaction with αA-crystallin, as the deletion of Arg 56 resulted in a decrease in the subunit exchange rate by ~18%, and the substitution for alanine reduced it further (~39%).

Studies show that αB-crystallins have the capacity to protect cells from oxidative damage and to inhibit apoptosis through the suppression of processing of the pro-apoptotic protein caspase-3 [50]. We found that the anti-oxidative activity was retained in αBΔ54–61 after the deletion of the 54–61 residues (Figure 7). ARPE -19 cells treated with αBΔ54–61-crystallin showed slightly higher anti-apoptotic activity than that demonstrated by αB-wt under similar experimental conditions (Figure 8). It is known that S19, S45, and S59 are major sites of phosphorylation in αB-crystallin [25]. It has been proposed that phosphorylation at these sites provides the switch point for conformational changes [39] and the modulation of apoptotic activity. Our observation suggests that S59, in phosphorylated or unphosphorylated form, likely has no role in the pro- or anti-apoptotic activity or the anti-oxidative property of αB-crystallin, contrary to the observation made during earlier studies [51]. Previously, the overexpression of the S59E mutant, a phosphorylation mimic, was found to promote vinblastine-induced apoptosis in breast cancer cells, while the S59A mutant, which cannot be phosphorylated, displayed a protective effect [52]. In another study, a pseudophosphorylation mutant, S19E, S45E, and S59E, did not show any anti-apoptotic activity [53]. Earlier investigations with αB-wt also concluded that interaction between crystallin and procaspase-3 leads to the prevention of caspase-3 maturation required for the apoptosis seen in oxidatively stressed cells [30,54]. Based on the data presented here, we propose that in addition to having a role in oligomerization, the 54–61 region in αB-crystallin puts some limit on the cytoprotective function of the protein because the deletion of the 54–61 region increased cell viability (Figure 6 and Figure 7C). Additional support for this view comes from examining the levels of EGR-1 in ARPE-19 cells treated with αBΔ54–61 or αB-wt and sodium iodate. As shown in Figure 9, the addition of αBΔ54–61-crystallin leads to the decreased expression of EGR-1 in sodium iodate-treated cells compared to cells treated with αB-wt. Increased EGR-1 expression is shown to be involved in lens epithelial apoptosis during oxidative stress [55]. The suppression of the EGR-1 level by crystallins suggests that sodium selenite-induced apoptosis involves EGR-1, and this transcription factor modulates the genes involved in apoptosis. It should be noted that an increase in the protective activity of αBΔ54–61 compared to that of αB-wt is not due to the difference in the uptake of the two proteins by ARPE-19 cells in culture because Western blot studies showed that the transduction of the two proteins was comparable (Figure 10).

It has been proposed that the phosphorylation of αB-crystallin provides the impetus for conformational changes [25,56]. The observation of oligomers with fewer subunits with increased chaperone activity, as seen with the 54–61 deletion mutant that has lost one of the major phosphorylation sites, S59, suggests that besides phosphorylation, other residues in the region do play a significant role in the structure function of αB-crystallin. While it appears that the phosphorylation of Ser59 is not required for anti-apoptotic activity or chaperone activity, additional studies are needed to uncover whether the phosphorylation of Ser 19 or Ser 45 was involved in the anti-oxidative or anti-apoptotic activity of αBΔ54–61 observed in ARPE-19 cells subjected to sodium selenite-mediated oxidative stress.

In summary, the deletion of the 54–61 sequence from αB-crystallin leads to smaller oligomers with higher chaperone and anti-apoptotic activity. Additionally, it appears that the phosphorylation of S59 is not required to enhance both activities.

## 4. Materials and Methods

### 4.1. Overexpression and Purification of Wild-Type and Mutant αB-Crystallins

The wild-type αB-crystallin and αBΔ54–61 mutant were expressed and purified as described previously with some modifications [20]. In brief, the wild-type protein and deletion mutants were expressed in *Escherichia coli* BL21(DE3)pLysS cells (Invitrogen Corp., Carlsbad, CA, USA) with IPTG (0.5 mM) induction. The cells (from 1 L culture) were lysed in 15 mL of lysis buffer (50 mM Tris-HCl, 2 mM EDTA, 0.1 M NaCl (pH 7.5)) containing 50 µL of protease inhibitor cocktail III (Calbiochem-EMD Millipore Corporation, Billerica, MA, USA), lysozyme (0.1 mg/mL) (Worthington, Lakewood, NJ, USA) and benzonase nuclease (25 units) (Sigma-Aldrich, St. Louis, MO, USA). The lysate was centrifuged at 18,000× *g* for one hour. The αB-crystallin or its mutant was precipitated by ammonium sulfate (45%) treatment from the supernatant fraction. The protein precipitate was resolubilized in 3 mL of PBS (phosphate buffer saline) and twice purified by gel filtration chromatography on a Hiload 16/60 Superdex G200 column (GE Healthcare Bio-Sciences Corp, Piscataway, NJ, USA). The fractions containing the purified crystallins were pooled, concentrated, and stored as 3 mg/mL aliquots at −80 °C for further use. The protein concentration was estimated by Bio-Rad protein assay, and the purity of the samples tested by SDS-PAGE was over 95% (Figure 1B).

### 4.2. Chaperone Assays

The chaperone activity of the wild-type αB-crystallin and deletion mutant were measured using lysozyme and luciferase (Promega, Madison, WI, USA) as the aggregation substrates. The assays were carried out on a Sepctramax i3 plate reader (Molecular Devices, San Jose, CA, USA). The light scattering at 360 nm was recorded as a function of time in the presence or absence of αB-wt or αBΔ54–61. Lysozyme (10 µM) aggregation assay was conducted at 37 °C in 0.25 mL PBS containing 2 mM DTT (GoldBio, St Louis, MO, USA) using 0–5 µM chaperone proteins. The luciferase (1 µM) aggregation was performed in PBS at 37 °C using 0–60 µM chaperone proteins. The relative chaperone efficiency of αB-wt and αBΔ54–61 against a substrate was compared by estimating the EC50 (effective chaperone protein concentration required to suppress the substrate protein aggregation by 50%) values. The EC50 values were calculated from the non-linear regression analysis obtained by plotting the % of substrate protein aggregation at the end of the assay for a known chaperone protein concentration. Sigmaplot V12.5 (Systat Software Inc., Palo Alto, CA, USA) dynamic curve fitting with four-parameter logistic curve function was used for non-linear curve fitting analysis.

### 4.3. Limited Proteolysis of Human αB-wt and αBΔ54–61 Using Trypsin

Human αB-wt and αBΔ54–61 (200 µg each) were mixed with 1 µg of sequencing-grade trypsin in PBS (pH 7.4) at a final reaction volume of 200 µL. Then, 20 µL aliquots were incubated at 37 °C for different time intervals ranging from 0 to 90 min, and the reaction was terminated by adding formic acid to a final concentration of 0.1%. Aliquots of the digested samples (~10 µg protein) were run on a 4–20% polyacrylamide gel and stained with Acqua stain (Bulldog Bio, Portsmouth, NH, USA). In parallel, the samples digested for 60 min were subjected to LC-MS analysis after diluting the peptide concentration to 1 pmol/µL. A portion of the diluted samples (18 µL) was loaded onto a C8 trap column (Thermo Scientific Pepmap100, 300 µm × 5 mm, 5 µm C8). Bound peptides were eluted from this trap column onto an 11 cm × 150 µm i.d. pulled-needle analytical column packed with C8 reversed-phase resin (Michrom Bioresources, Auburn, CA, USA). Peptides were separated and eluted from the analytical column with a gradient of acetonitrile at 600 nL/min as follows: initial conditions 20% B (A: 0.1% formic acid in water; B: 99.9% acetonitrile, 0.1% FA), hold at 20% B for 4 min, gradient from 20–60% B over 6 min, ramp to 90% B over 1 min, hold at 90% B for 10 min, ramp back to (2 min) and hold at (4 min) initial condition. The total run time was 30 min. The Proxeon Easy nLC HPLC system attached to an LTQ Orbitrap XL mass spectrometer was used for the analysis. High-resolution (100,000 resolution, 1 microscan, 5e5 AGC) FTMS data were acquired of the eluting fragments. Qual browser (V2.0.7) was used to deconvolute multi-charge-state FTMS data using the FT Programs to extract all applications (extract MW, 100 K resolution, s/*n* >2).

### 4.4. Chaperone–Substrate Complex Studies

The extent of the complex formation by the wild-type and deletion mutant αB-crystallin when incubated with CS at 43 °C was examined by light scattering analysis at different time intervals. Samples with a chaperone-to-substrate ratio of 1:2 were incubated at 43 °C for the desired time before evaluation. The reaction mixtures of either αB-wt or αBΔ54–61 with citrate synthase were passed through a TSK G5000PW_XL_ size exclusion column (Tosoh Bioscience, King of Prussia, PA, USA) connected to a Shimadzu HPLC system with UV and refractive index detectors and coupled to a static multi-angle laser light scattering (MALS) and quasi-elastic light scattering system (Wyatt Technology Corporation, Santa Barbara, CA, USA). The molar mass, size, and polydispersity were determined using ASTRA 6.1 software package (Wyatt Technology).

### 4.5. Cytoprotective Effect of αB-wt and αBΔ54–61 in ARPE-19 Cells

Pre-authenticated ARPE-19 cells (ATCC, Manassas, VA, USA) were seeded in 96-well plates in Dulbecco’s modified Eagle’s medium (DMEM) + 5% FBS and maintained in an incubator at 37 °C, set at 5% CO_2_ flow until the cells were 60−70% confluent. The cells were then serum starved for 2 h at 37 °C, after which they were treated with 0.5, 1, or 2.5 μM of either αB-wt or αBΔ54–61 proteins. Cytotoxicity was induced by adding sodium iodate (SI, 7.5 mM final concentration) to cells in serum-free DMEM. After incubation for 24 h, the EarlyTox Cell Integrity assay kit (Molecular Devices, San Jose, CA, USA) was used to detect and differentiate between live and dead cells. The extent of dead cells was estimated by measuring the fluorescence intensity at 541 nm (green) in a Molecular Devices plate reader Spectramax I3. Empirically, cells with average fluorescence intensities of ≥1500 units at 541 nm were considered dead. The representative data shown are the average readings from six wells per sample. Data were analyzed by the ANOVA single factor test for statistical significance. All experiments were repeated three times.

### 4.6. Cell Viability Assay

Authenticated ARPE-19 cells were cultured on a 96-well plate and treated with 7.5 mM of sodium iodate and αB-wt or αBΔ54–61. After 24 h, the number of viable cells was quantified using CellTiter Glo 2.0 (Promega, Madison, WI, USA) according to the manufacturer’s protocol. The data shown are an average of six analyses performed on images captured from different wells.

### 4.7. Anti-Oxidative Potential of αB-wt and αBΔ54–61

To evaluate the ROS scavenging ability of αB-crystallins on ARPE-19 cells, 2′,7′-Dichlorofluorescin diacetate (DCFH-DA) assay was performed. Authenticated ARPE-19 cells cultured on a 96-well plate were treated with 7.5 mM of sodium iodate and αB-wt or αBΔ54–61. After 24 h, oxidatively stressed cells were identified using DCFH-DA (MilliporeSigma, St. Louis, MO, USA) as per the manufacturer’s protocol and imaged on the EVOS FL Auto2 imaging system. The data shown are an average of six analyses performed on images captured from different wells.

### 4.8. Effect of Transduced αB-wt and αBΔ54–61 on Caspase Activation in ARPE-19 Cells

Pre-authenticated ARPE-19 cells were treated with 7.5 mM of SI and αB-wt or αBΔ54–61, and the caspase activation was determined after 24 h using NucView 488 Caspase-3 assay kit (Biotium, Inc.) according to the manufacturer’s protocol. After 24 h, fluorescence images of cells stained with the caspase-3/7 substrate (NucView 488; Biotium, Inc.) were collected, and the number of cells with activated caspase-3/7 was also determined. The data shown are an average of six analyses performed on images captured from different wells.

### 4.9. Regulatory Effect of αB-wt and αBΔ54–61 on Apoptotic Cascade Components in ARPE-19 Cells

To compare the WT and deletion mutant’s anti-apoptotic activities, we examined the expression of apoptotic cascade components in ARPE-19 cells simultaneously treated with 7.5 mM SI and with 1 μM chaperone proteins. We focused our attention by examining the protein levels of early growth response protein-1 (EGR-1; Santa Cruz Biotechnology, Dallas, TX, USA; cat. no. sc-515830; 1:3000 dilution), cytochrome c oxidase I (COX-I; Santa Cruz Biotechnology; cat. no. sc-19998; 1:2000 dilution), and B-cell chronic lymphoma 2 (Bcl-2; Santa Cruz Biotechnology; cat. no. sc-7382; 1:2000 dilution) using an immunoblot analysis of treated cell extracts. After 24 h, the treated cells were washed three times with PBS; the cells were lysed, and the lysates were analyzed by Western blot using respective antibody dilutions. Cell lysates were run on 4–20% SDS-PAGE gel (Genscript, Piscataway, NJ, USA) at 120 v for 90 min. Proteins were transferred to the PVDF membrane (0.22µ) at 25 V for 60 min using BioRad Semi-Dry transfer apparatus. The membrane was then incubated with primary protein-specific antibodies overnight at 4 °C. The membranes were incubated with the ScanLater Eu-labeled anti-rabbit or anti-mouse secondary antibodies (Molecular Devices) for 2 h. Blots were washed, dried, and scanned using a Spectramax i3 plate reader equipped with ScanLater Western Blot Detection Cartridge. The experiments were repeated three times.

### 4.10. Relative Amounts of Protein Transduced into the ARPE-19 Cells

ARPE-19 cells cultured on a 6-well plate were incubated with 0.5 and 1 µM of αB-wt and αBΔ54–61 in basal medium. After 24 h, the medium was removed; the treated cells were washed three times with sterile DPBS, and the cells were lysed. The lysate was analyzed by Western blot using an αB-crystallin-specific antibody (Enzo Life Science, Inc., Farmingdale, NY, USA cat. no. ADI-SPA-223-F, 1:5000 dilution), and β-actin (Sigma-Aldrich, cat. no. P8340, 1:2000 dilution) acted as an internal control. The amount of transduced proteins was normalized to cellular β-actin protein levels. Experiments were repeated three times.

### 4.11. Statistical Analysis

Differences between the groups were assessed using a one-way ANOVA single factor test for statistical significance using SPSS for Windows (version 21, IBM Corporation, New York, NY, USA) and SigmaPlot (version 12.5) graphics software. One-way ANOVA yielded significant results, and post hoc testing was performed for inter-group comparisons using the least significant difference test. Values corresponding to *p* < 0.05 were considered statistically significant and are denoted by distinct symbols in the tables and figures. The values are expressed as the mean ± standard deviation of multiple readings.

## Figures and Tables

**Table 1:** MALS analysis of chaperone protein and CS incubation mixtures.

**αB-wt + CS**						
	**Peak 1** **Citrate Synthase**		**Peak 2 ** **Crystallin or Complex**		**Peak 3 ** **Complex**	
	**(11.5–12.3 min)**		**(9.9–10.8 min)**		**(7.8–9.8 min)**	
**Incubation time (min)**	**Average Mw** **(kDa)**	**Rh** **(nm)**	**Average Mw** **(kDa)**	**Rh** **(nm)**	**Average Mw** **(kDa)**	**Rh** **(nm)**
0	117 (± 0.5%)	3.95 ± 0.17	612 (± 0.2%)	7.23 ± 0.23	ND	ND
40	126 (± 0.2%)	4.10 ± 0.17	588 (± 0.2%)	7.20 ± 0.23	ND	ND
80	127 (± 0.2%)	4.30 ± 0.18	621 (± 0.1%)	7.39 ± 0.24	ND	ND
**αBΔ54–61 + CS**						
	**Peak 1** **Citrate Synthase**		**Peak 2 ** **Crystallin or Complex**		**Peak 3 ** **Complex**	
	**(11.5–12.3 min)**		**(10.3–11.0 min)**		**(7.8–9.8 min)**	
**Incubation time (min)**	**Average Mw** **(kDa)**	**Rh** **(nm)**	**Average Mw** **(kDa)**	**Rh** **(nm)**	**Average Mw** **(kDa)**	**Rh** **(nm)**
0	110 (± 0.5%)	3.92 ± 0.18	306 (± 0.4%)	5.3 ± 0.2	ND	ND
40	174 (± 0.6%)	6.41 ± 0.25	784 (± 0.5%)	12.0 ± 0.4	8105 (± 0.4%)	16.9 ± 0.5
80	333 (± 0.5%)	11.9 ± 0.40	1725 (± 0.4%)	18.9 ± 0.5	21,000 (± 0.4%)	21.9 ± 0.6

ND - No detectable peaks for analysis.

## Data Availability

Not applicable.
